# The Rise of Personalized Medicine in Heart Failure Management: A Narrative Review

**DOI:** 10.7759/cureus.83731

**Published:** 2025-05-08

**Authors:** Elizabeth Caroline Palaparthi, Palle Aditya Reddy, Tanvi Padala, Kalahasthi Sri Venkata Mahi Karthika, Reshika Paka, Vignesh Ami Reddy, Shirin Ayub, Vanga Khyati Sri, Vubasaram Rebanth Nandan, Prashanth Kumar Patnaik, Tambi Medabala, Suresh Babu Sayana

**Affiliations:** 1 Department of Internal Medicine, Shasta Regional Medical Center - Prime Healthcare, Redding, USA; 2 Department of Pharmacology, RVM Institute of Medical Sciences and Research Center, Laxmakkapally, IND; 3 Department of Pharmacology, Bhaskar Medical College, Moinabad, IND; 4 Department of Pharmacology, RVM Institute of Medical Sciences and Research, Laxmakkapally, IND; 5 Department of Physiology, Sports Authority of India, NSNIS Patiala, Patiala, IND; 6 Department of Pharmacology, Government Medical College and General Hospital, Bhadradri Kothagudem, IND

**Keywords:** artificial intelligence (ai), biomarkers, digital health, gene therapy, heart failure (hf), multi-omics, personalized medicine, pharmacogenomics

## Abstract

Managing heart failure (HF) presents ongoing challenges due to the varied nature of the condition among individuals. Recent shifts toward personalized care aim to move beyond standard treatments by considering the unique characteristics of each patient. This review brings together current ideas and advancements that could shape a more tailored approach to HF, offering insights into how patient-specific strategies might improve the care. However, fully embracing this approach requires overcoming several hurdles to ensure these innovations are practical and widely available.

## Introduction and background

Heart failure

Heart failure (HF) is a complex clinical syndrome characterized by the heart's inability to pump sufficient blood to meet the body's metabolic needs or demands, either due to impaired contraction (systolic dysfunction) or inadequate filling (diastolic dysfunction) [1]. HF is commonly categorized into three types based on left ventricular ejection fraction (LVEF), which indicates how well the heart pumps blood. Heart failure with reduced ejection fraction (HFrEF), also known as systolic heart failure, occurs when the heart’s ability to contract is impaired, leading to a reduced LVEF (typically less than 40%). This type is most commonly caused by coronary artery disease, myocardial infarction, or dilated cardiomyopathy. The heart’s inability to pump efficiently leads to symptoms such as shortness of breath, fatigue, and fluid retention. Heart failure with preserved ejection fraction (HFpEF), or diastolic HF, involves a stiffened heart muscle that cannot relax properly during diastole, impairing the heart’s ability to fill with blood despite a normal or near-normal LVEF (usually above 50%). HFpEF is most often linked to conditions like hypertension, diabetes, and obesity, and it is more prevalent in older adults. Lastly, heart failure with mildly reduced ejection fraction (HFmrEF) is an intermediate subtype characterized by an LVEF ranging from 40% to 50%. Patients with HFmrEF may experience symptoms similar to those with HFrEF or HFpEF, and this subtype is thought to involve a combination of systolic and diastolic dysfunction [2]. These subtypes display unique molecular and pathological traits, leading to varied clinical courses and different responses to therapy. This heterogeneity stresses more precise diagnostic and therapeutic strategies. HF poses a significant global health burden and affects more than 64 million individuals worldwide. Youth-onset HF is increasingly linked to rising rates of obesity, metabolic syndrome (MetS), and diabetes in adolescents and young adults. Obesity causes structural and functional changes in the heart, including left ventricular hypertrophy and diastolic dysfunction, both of which are early indicators of HFpEF. MetS, driven by poor diet and physical inactivity, is often diagnosed in younger populations and increases the risk of heart failure. The growing incidence of type 2 diabetes among youth, along with related cardiovascular issues, further contributes to the emergence of HF [3]. HF greatly contributes to morbidity, mortality, and healthcare burden [4]. HF is particularly heterogeneous in its clinical presentation, prognosis, and response to treatment, underlining the complexity of managing the condition. Comorbid conditions such as diabetes mellitus and chronic kidney disease are usually more prevalent among HF patients. These comorbidities greatly affect disease progression, management strategies, and clinical outcomes. Such comorbidities often complicate clinical decision-making by posing challenges in selecting optimal treatments. These indicate the need for individualized care in HF management [5].

Genetic and epigenetic variability

Genetic and epigenetic differences among the patients significantly influence HF susceptibility, its progression and responses to pharmacological and nonpharmacological treatments. Variants in genes such as the beta-1 adrenergic receptor (ADRB1), angiotensin-converting enzyme (ACE), cytochrome P450 2D6 (CYP2D6), and solute carrier organic anion transporter family member 1B1 (SLCO1B1) illustrate the role of genetic polymorphisms in therapeutic responses and adverse effects. It emphasizes the potential of pharmacogenomics to refine treatment selection [6-8].

Limitations of conventional "one-size-fits-all" treatments

Traditional HF management approaches typically follow a standardized therapeutic regimen based on large population data, often overlooking individual patient variability. This error or mistake can lead to suboptimal outcomes, treatment resistance, and increased rates of adverse events. Consequently, there is growing awareness of the limitations inherent in conventional treatment strategies, prompting a shift towards precision-based medicine [9].

Definition and scope of personalized medicine (PM)

PM, also known as precision medicine, can tailor medical treatment to the unique characteristics of each patient. This approach integrates various possible characteristics of the patients, like genetic, environmental, lifestyle, and clinical information, to inform prevention, diagnosis, and treatment decisions. PM aims to improve the clinical efficacy, minimize adverse effects, and enhance overall patient outcomes through more targeted interventions [10]. Integrating PM into HF management can significantly transform patient care by enhancing risk stratification., It enables precise therapeutic targeting and optimizes clinical outcomes. The personalized approach could alleviate the disease burden, improve quality of life, decrease hospitalizations, and boost cost-effectiveness in HF treatments [10,11].

This narrative review evaluates the current evidence on PM approaches in HF, highlighting advancements in the distinct molecular diagnostics, pharmacogenomics, and technology-driven interventions.

## Review

Database search strategy and selection

A search strategy was developed and executed across multiple scientific databases to ensure coverage of the literature on PM in HF. Databases searched included PubMed/MEDLINE and Google Scholar. The literature search spanned publications from January 2000 to March 2025, capturing relevant advancements over two decades. The following keywords were employed: "Heart Failure" OR "heart failure" OR "HF" OR "congestive heart failure") AND ("Personalized Medicine" OR "precision medicine" OR "personalised medicine" OR "tailored therapy") AND ("biomarkers" OR "genomics" OR "pharmacogenomics" OR "proteomics" OR "transcriptomics" OR "multi-omics" OR "artificial intelligence" OR "machine learning" OR "digital health" OR "wearables". Peer-reviewed original articles, reviews, meta-analyses, and guidelines published between January 2000 and March 2025 were screened, focusing specifically on PM approaches in HF involving biomarkers, genetics, epigenetics, omics-based data, AI, digital health, or wearable technologies.

Molecular diagnostics in HF

HF involves a complex pathophysiological mechanism. It presents substantial clinical challenges that require timely diagnosis and accurate prognosis. Recent advances in molecular diagnostics of HF have significantly transformed diagnosis, risk-stratification, and management. Critical examination of the current evidence highlights clear trends, existing gaps, contradictions, and opportunities for clinical translation.

Current Evidence and Critical Synthesis

Established biomarkers: Traditional natriuretic peptides (B-type natriuretic peptide (BNP) and N-terminal pro-BNP (NT-proBNP)) remain cornerstone biomarkers due to robust predictive accuracy in diagnosing acute decompensation and stratifying mortality risk [12]. Furthermore, recent systematic evaluations (ESC Guidelines, 2023) continue to affirm their clinical utility, highlighting NT-proBNP’s superiority due to its central role as a biomarker for guiding therapy and monitoring treatment efficacy of post-acute HF of HFpEF and HFmrEF subtypes [13].

Emerging biomarkers: Newer biomarkers such as Galectin-3 and soluble suppression of tumorigenicity 2 (sST2) represent fibrosis and myocardial stress pathways, respectively. Galectin-3 elevation correlates strongly with fibrosis progression, significantly predicting hospitalizations and mortality, especially in HFpEF populations. Contrastingly, sST2 uniquely captures acute myocardial stress, offering incremental prognostic value over NT-proBNP alone. Recent evidence further supports the predictive accuracy of combined biomarker panels (NT-proBNP, sST2, and Galectin-3), advocating for a multimodal biomarker approach rather than reliance on single markers [14]. Nevertheless, recent studies have highlighted the clinical challenges posed by the lack of standardized Galectin-3 cutoffs, leading to inconsistencies in patient classification and treatment decisions [15].

The quality of current biomarker evidence is mixed. While NT-proBNP holds high-quality evidence supported by extensive clinical validation, evidence supporting emerging biomarkers like Galectin-3, sST2, and miRNAs remains moderate, constrained by limited prospective trials. Recently published meta-analyses and systematic reviews reinforce their prognostic utility but simultaneously call for more rigorous methodological standardization and larger prospective validations [13,14,16-18].

Novel Insights: MicroRNAs and Multi-Omic Integration

A rapidly expanding area of novel diagnostics includes circulating microRNAs (miRNAs), small non-coding RNAs involved in gene regulation, which are emerging as powerful diagnostic and prognostic tools. For example, miR-21-5p, miR-30a-3p, miR-30a-5p, miR-155-5p, miR-216a, and miR-217 have shown promising results in differentiating HF phenotypes and identifying early myocardial remodeling before traditional biomarkers rise [16]. This positions miRNAs as potentially transformative tools for preclinical detection and precise phenotypic differentiation. Furthermore, integration of miRNA data with transcriptomic and proteomic platforms presents an unprecedented potential for personalized risk stratification, bridging traditional biomarkers and genomic data for more precise, individually tailored interventions [16].

Natriuretic peptides, particularly NT-proBNP, remain at the forefront of HF diagnosis and management, supported by strong clinical evidence. Additional biomarkers such as Galectin-3 and sST2 provide valuable prognostic information, particularly in identifying patients with ongoing fibrosis or inflammation. However, their use requires careful interpretation due to variability across different patient groups. Circulating miRNAs are also emerging as potential biomarkers, offering early insights into disease progression and phenotyping, though further validation is needed before they can be routinely applied in clinical practice. Current recommendations encourage a combined approach, using established and emerging biomarkers to improve risk assessment and support more personalized treatment strategies in HF care; details are summarized in Table 1.

**Table 1:** Summary of Biomarkers in Heart Failure: Roles, Utility, and Limitations This table was created by the authors based on the synthesis of current evidence from references [13–16]. HF: Heart failure; BNP: B-type natriuretic peptide; NT-proBNP: N-terminal pro-BNP

Biomarker	Pathophysiological Role	Clinical Utility	Limitations
NT-proBNP/BNP [13]	Ventricular stretch, volume overload	Diagnosis, risk stratification, prognosis, therapy guidance	Influenced by age, renal function, and obesity
Galectin-3 (Gal-3) [14]	Fibrosis, inflammation	Prognosis, risk stratification in HF	Non-cardiac specificity (renal, cancer); variability across studies
sST2 (soluble ST2) [13,14,16–18]	Myocardial stress, fibrosis	Prognosis, risk stratification, and potential therapy guidance	No reduction in mortality as a standalone marker
microRNAs (miRNAs) [16]	Gene regulation, myocardial remodeling, fibrosis	Early diagnosis, HF phenotyping, risk stratification	Lack of standardization, small sample sizes

Gaps, Contradictions, and Challenges

Despite promising developments, substantial gaps in biomarkers persist. Critical evaluation indicates a lack of universal threshold values for biomarkers like Galectin-3 and sST2 yet creates clinical ambiguity [18]. On the other hand, contradictions arise in biomarker predictive value across diverse patient populations, notably influenced by comorbidities (renal insufficiency, obesity) affecting biomarker accuracy. Additionally, although miRNAs are promising, inconsistencies in their quantification methods and the absence of standard reference ranges limit immediate clinical translation [19]. An overarching challenge remains the validation of biomarker panels in large-scale, prospective trials. Current evidence predominantly comes from observational cohorts or secondary analyses, necessitating robust randomized controlled trials (RCTs) to confirm the added clinical value before routine adoption [18,19].

Future Clinical and Research Directions

Future research might prioritize standardized biomarker thresholds. Addressing heterogeneity in patient populations and conducting robust, prospective multicenter trials is very much required. Clinical translation efforts may focus on creating practical biomarker-guided algorithms that could demonstrate cost-effectiveness and clinical utility. Integrating molecular biomarkers into digital and pharmacogenomic platforms might enhance personalized HF management. Molecular diagnostics represent a pivotal element in personalized HF management. Established biomarkers remain clinically essential, while emerging molecular markers and multi-omic approaches offer transformative potential for benefiting HF patients. However, clinical translation demands robust evidence generation through methodologically sound trials or research. These elements may address existing gaps in standardization, and clinical validation will decisively position molecular diagnostics at the core of personalized HF care.

Pharmacogenomics in HF

Pharmacogenomics is the study of how genetic variations influence drug responses, and it has gained increasing attention in HF management. Tailoring medication choices and dosages based on individual genetic profiles promises improved therapeutic outcomes and reduced adverse drug reactions. A critical analysis of current evidence reveals significant opportunities, existing contradictions, and the necessary direction for future research [20-23].

Current Evidence

Genetic determinants of drug response: Polymorphisms in β-adrenergic receptors ((adrenoceptor beta 2 (ADRB1) and adrenoceptor beta 2 (ADRB2)), angiotensin-converting enzyme (ACE), and drug-metabolizing enzymes like cytochrome P450 family-2 subfamily D member 6 (CYP2D6) significantly affect the pharmacodynamics and pharmacokinetics in HF medications, including beta-blockers, ACE inhibitors, and angiotensin receptor blockers (ARBs). For instance, in the ADRB1, Arg389Gly polymorphism has consistently demonstrated differential responsiveness to beta-blocker therapy, with Arg389 allele carriers exhibiting better survival rates and reduced hospitalizations compared to Gly389 carriers [24]. Similarly, CYP2D6 polymorphisms are crucial for drugs such as metoprolol and carvedilol. Poor metabolizers experience higher drug concentrations, potentially leading to adverse events like bradycardia or hypotension, while ultra-rapid metabolizers may experience inadequate therapeutic effects, compromising clinical outcomes [6-8].

The current strength of evidence for pharmacogenomic applications in HF management can be classified as moderate. Among the more robust findings, genetic variants like ADRB1 polymorphisms exhibit moderate to high evidentiary quality, having been substantiated through multiple cohort-based investigations and retrospective datasets. Conversely, the supporting data for ACE polymorphisms, along with emerging polygenic risk frameworks, remain in early developmental stages. Much of this evidence is derived from observational and limited-sample studies, thus warranting cautious interpretation. Comprehensive analyses, including recent systematic reviews, emphasize a pressing need for expansive, multicenter randomized trials to rigorously establish both clinical relevance and translational value before such tools are considered suitable for standard clinical adoption [6-8].

Novel Insights: Polygenic Risk Scores and Integrative Approaches

Recent advances extend beyond single-gene assessments, moving towards polygenic risk scores that integrate multiple genetic variants to predict therapeutic responses and risks more accurately. Novel integrative frameworks using genome-wide association studies and next-generation sequencing data have emerged, aiming to individualize HF therapy comprehensively. Such models incorporate genetic risk factors alongside clinical and biomarker data, leveraging AI-driven algorithms to identify patient-specific drug response patterns [25,26].

Table 2 explains key highlights from the pharmacogenomic evidence in HF which show that genetic variations, particularly in ADRB1 and CYP2D6, significantly determine individual responses to beta-blocker therapy. While ADRB1 Arg389 carriers often show better outcomes, CYP2D6 poor metabolizers are at risk of adverse effects due to higher drug concentrations. ACE gene polymorphisms also influence response to ACE inhibitors, though findings remain inconsistent across populations. More recent approaches, like polygenic risk scores, aim to integrate multiple genetic markers to predict drug responses better, offering a broader, more precise strategy for personalized HF management, though further validation is required.

**Table 2:** Summary of Pharmacogenomic Variants Relevant to Heart Failure Management The table created by the authors is based on a synthesis of current evidence from references [6–8,24,26] GWAS: Genome-wide association study; CPIC: Clinical Pharmacogenetics Implementation Consortium

Genetic Variant	Gene(s)	Drug(s) Affected	Clinical Impact	Technology/Platform to Detect
ADRB1 Arg389Gly [24]	ADRB1	Beta-blockers	Arg389 allele enhances beta-blocker response; Gly389 reduces responsiveness​	PCR-based genotyping; GWAS
CYP2D6 variants [6–8]	CYP2D6	Beta-blockers, antidepressants	Poor metabolisers show higher drug levels (risk of adverse effects); ultra-rapid metabolizers show lower efficacy​	PCR-based genotyping; CPIC guidelines
ACE polymorphism [6–8]	ACE	ACE inhibitors	D-allele associated with differential response and prognosis; mixed findings across studies	PCR-based genotyping
VKORC1 & CYP2C9 [8]	VKORC1, CYP2C9	Warfarin	Influence dosing requirements; reduce bleeding risk with genotype-guided dosing​	PCR-based genotyping; clinical pharmacogenetic models
SLCO1B1 [8]	SLCO1B1	Statins	Variants increase the risk of statin-induced myopathy​	PCR-based genotyping
Polygenic risk scores (PRS) [26]	Multiple genes	Beta-blockers, ACE inhibitors	Integrates multiple variants to predict drug efficacy/survival; emerging as superior to single-gene markers​	GWAS, machine learning algorithms

Gaps, Contradictions, and Challenges

Although the existing body of evidence is encouraging, several critical gaps persist. One major inconsistency lies in the unreliable replication of genetic associations across different ethnic populations and healthcare contexts. The overrepresentation of individuals of European ancestry in most studies restricts broader applicability and raises ethical dilemmas related to the fairness of medical care distribution. While findings on ADRB1 polymorphisms tend to be consistent, the data on ACE polymorphisms remain conflicting; various investigations report differing degrees of therapeutic advantage, thereby limiting their immediate relevance in personalized pharmacotherapy. Additionally, a substantial barrier exists in translating genetic findings into practical clinical protocols. Numerous studies in this domain are observational in nature, and rigorously designed RCTs confirming the clinical validity of genotype-informed treatment remain limited. Similarly, economic analyses substantiating the cost-efficiency of such interventions are minimal, posing further challenges to their integration into routine clinical practice [20].

Future Clinical and Research Directions

Future research must prioritize large-scale, prospective clinical trials assessing genotype-guided prescribing's clinical outcomes and economic feasibility. Studies should deliberately include ethnically diverse populations to improve generalizability. Furthermore, integrating pharmacogenomic data with electronic health records (EHRs) and clinical decision-support tools represents a vital step toward practical implementation. Research into patient and clinician acceptance, educational initiatives, and ethical considerations must parallel clinical validation efforts.

Pharmacogenomics holds substantial promise for transforming HF therapy through genetically tailored treatment paradigms. Although robust evidence supports certain genetic markers (e.g., ADRB1 polymorphisms), critical gaps in standardization, evidence validation, and clinical translation remain. Addressing these challenges through rigorous, diverse, and integrative research is essential for pharmacogenomics to move from theoretical promise to clinical reality, significantly improving personalized patient care in HF.

Technology-driven interventions in HF

Digital health innovations have recently gained momentum in reshaping the clinical management of HF. Tools such as wearable biosensors, AI-driven analytics, mobile health (mHealth) applications, and remote surveillance technologies are no longer peripheral; they are becoming integral to enabling timely detection, facilitating early interventions, and encouraging patient engagement in daily care. As this field rapidly evolves, critical evaluations of the evidence reveal not only substantial benefits but also contradictions and persistent uncertainties. These complexities underscore the necessity for rigorous inquiry and point toward research priorities aimed at enhancing the clinical assimilation of these technologies [27].

Current Evidence and Critical Synthesis

Remote monitoring and wearable technologies: The integration of technologies like implantable pulmonary artery pressure monitors, such as CardioMEMS, and commercially available wearable biosensors, including ECG-enabled devices like the Apple Watch and Fitbit, has yielded measurable clinical value. Notably, implantable systems contribute to a marked decline in HF-related hospital admissions. This benefit is primarily attributed to their capacity for early recognition of hemodynamic deviations, allowing intervention well before clinical symptoms become apparent, thereby preventing decompensation [28]. Non-invasive wearable technologies, capable of persistently monitoring physiological parameters such as heart rate, activity levels, and rhythm abnormalities, offer the prospect of facilitating timely, preventive medical responses. Yet, despite this potential, the current body of evidence remains inconclusive. While certain investigations document meaningful gains in clinical outcomes, others raise concerns regarding the reliability of data captured and the consistency of patient engagement with such devices. These disparities underscore the need for more rigorous, context-specific evaluations before widespread clinical endorsement [28,29].

AI and predictive analytics: AI-enhanced prediction models synthesizing clinical variables, genomic profiles, biomarkers, and sensor-derived metrics have gained traction as transformative tools for anticipating HF decompensation. Trials employing AI-guided risk stratification have demonstrated superior accuracy in flagging high-risk individuals, resulting in notable reductions in hospital admissions and mortality compared to traditional surveillance approaches. Nevertheless, despite these encouraging outcomes, the clinical deployment of such technologies remains limited. The transition from research to real-world practice is hindered by technical barriers, including fragmented data systems and a lack of uniform standards, as well as ethical challenges related to algorithmic opacity and potential bias [25,30,31].

The quality and strength of evidence for technology-driven interventions vary widely. Implantable sensors, notably CardioMEMS, present high-quality evidence supported by multiple prospective randomized controlled trials that demonstrate clear clinical benefits. In contrast, evidence for wearable devices, mobile applications, and AI-driven interventions remains moderate, characterized primarily by observational studies or pilot trials with heterogeneous methodologies. Recent systematic reviews and meta-analyses underscore the urgent need for large-scale randomized trials to confirm these promising preliminary outcomes conclusively [28].

Novel Insights: Digital Therapeutics and Personalized Feedback

Digital health advancements have evolved beyond passive monitoring, giving rise to digital therapeutics (DTx) that deliver individualized interventions such as behavior modification strategies, adherence prompts, nutritional guidance, and direct communication channels between patients and providers. Integrating personalized mobile platforms with real-time sensor data has shown promising effects, particularly in supporting sustained compliance with lifestyle changes and pharmacologic regimens, thereby mitigating the risk of HF exacerbations. Additionally, the synergistic use of AI-driven analytics and virtual care modalities enables anticipatory management, fostering greater patient autonomy and deepening their engagement in day-to-day disease control [32].

The current evidence highlights that implantable sensors like CardioMEMS and multi-parametric algorithms such as Triage HF have stronger clinical backing, showing clear benefits in reducing hospitalizations and improving patient outcomes. AI-driven predictive models are increasingly embedded within implantable devices, enhancing risk stratification and enabling early interventions. However, digital health tools like telemedicine platforms and mobile health apps, though helpful for improving self-care and patient engagement, still require more robust evidence to confirm their clinical effectiveness; details are summarized in Table 3.

**Table 3:** Summary of Technology-Driven Interventions in Heart Failure Management The table was compiled by the authors based on synthesis of current evidence from references [27,28,31-33] HF: Heart failure

Technology/Intervention	Device/Platform	Functionality Monitored	Clinical Outcomes Observed
Pulmonary artery pressure monitoring [28]	CardioMEMS	Pulmonary artery pressure	48% reduction in hospitalizations; improved QoL
Wearable devices [33]	Apple Watch, Fitbit, ActiGraph	ECG, HR, oxygen saturation, physical activity	Improved self-care behaviors; mixed evidence for hospitalization reduction
AI-driven predictive algorithms [31,32]	Heart-Logic, SELENE-HF, Triage HF	Multi-parametric (HR, impedance, arrhythmias, activity)	Early warnings for decompensation, reduced hospitalizations
Telemedicine & mobile health [27]	Telehealth platforms, Health apps	Symptom reporting, vitals, medication adherence, and patient education	Improved self-care, no major impact on clinical outcomes

Gaps, Contradictions, and Challenges

Despite the momentum surrounding digital health, several unresolved tensions and structural gaps continue to hinder its full potential. Remarkably, findings from clinical trials assessing the efficacy of wearable sensors remain inconsistent, particularly among older adults and individuals with multiple comorbidities, groups for whom digital engagement and adherence are often compromised. In parallel, unresolved issues regarding data protection, patient confidentiality, and the ethical ramifications of persistent surveillance raise significant concerns. Equally pressing are the risks of exacerbating digital inequities due to disparities in access, affordability, and infrastructure. Compounding these barriers is a regulatory environment that has yet to evolve at the pace of technological advancement, thereby restricting the broader integration of emerging digital therapeutic models into routine care [29].

Future Clinical and Research Directions

Advancing digital health integration in HF care requires rigorous randomized controlled trials to evaluate clinical efficacy, patient engagement, and long-term cost-effectiveness. Addressing digital inequities through inclusive access and clear regulatory frameworks is essential for ethical implementation. Future research must also prioritize AI transparency, minimize algorithmic bias, and validate tools across diverse populations. Standardizing digital data integration into EHRs is crucial for informed, real-time decision-making. While implantable devices demonstrate proven benefits, further validation is needed for wearables, mHealth platforms, and predictive AI. Realizing the full potential of these innovations hinges on robust validation, standardization, and equitable deployment to improve outcomes and efficiency in HF management [27].

Challenges and gaps in PM for HF management

Although PM holds the potential to revolutionize HF care through precision diagnostics, individualized treatment strategies, and enhanced clinical outcomes, its widespread clinical adoption remains constrained by persistent challenges and unresolved gaps. A thorough analysis of these limitations uncovers structural inconsistencies and system-level hurdles that demand focused intervention. Addressing these concerns will require coordinated efforts from healthcare providers, researchers, policymakers, and broader stakeholders committed to advancing equitable and effective implementation [34].

Evidence Gaps and Methodological Limitations

A central limitation within the personalized HF research landscape is the lack of robust evidence from large-scale RCTs. Much of the current literature is grounded in observational designs, retrospective cohorts, or exploratory pilot studies, particularly in domains such as pharmacogenomics and digital therapeutics. These methodological constraints introduce risks of bias, confounding variables, and limited reproducibility, thereby complicating the formulation of conclusive clinical guidance. The absence of prospective validation studies remains a critical barrier, significantly weakening the strength of clinical recommendations. This underscores an urgent imperative for well-designed, adequately powered RCTs to rigorously evaluate safety and efficacy prior to broader clinical implementation [35].

Standardization and Clinical Implementation Challenges

The absence of standardized biomarker thresholds, genetic variant interpretations, and digital data integration protocols remains a substantial barrier to practical clinical implementation. For instance, molecular diagnostics (e.g., Galectin-3, soluble ST2) and pharmacogenomic testing (e.g., ADRB1 variants) suffer from inconsistent reference ranges and lack universally accepted guidelines, resulting in significant clinical ambiguity [18]. Similarly, wearable technology and AI-driven platforms lack consistent data standards and interoperability, complicating their integration into routine EHR systems. This fragmentation significantly diminishes clinicians' and healthcare institutions' confidence in broadly adopting PM technologies [27].

Ethical, Legal, and Social Issues (ELSI)

Integrating genetic testing and continuous digital monitoring in HF management introduces substantial ethical and legal concerns. Genetic data privacy and patient autonomy remain unresolved, as current policies inadequately address genetic data ownership, protection, and the potential for discrimination. Similarly, remote monitoring raises significant ethical concerns related to surveillance, consent, and patient autonomy, particularly among vulnerable populations (elderly, cognitively impaired, socioeconomically disadvantaged groups) who face higher barriers to informed participation. These unresolved ethical and social concerns form critical obstacles, requiring clear regulatory frameworks and thoughtful policy-making that balance technological innovation with ethical responsibility [36].

Economic and Accessibility Disparities

Economic constraints represent a significant barrier to the widespread adoption of PM in HF care. Although clinical advancements in implantable monitoring systems and pharmacogenomic applications demonstrate promising outcomes, the substantial initial investment often limits their accessibility. Existing cost-effectiveness data are sparse, and early findings suggest considerable variation in economic viability across healthcare infrastructures and socioeconomic strata. These financial barriers are especially pronounced in under-resourced environments, where the cost burden may exacerbate health inequities. Paradoxically, rather than promoting health equity, the current implementation landscape risks reinforcing disparities, which PM seeks to address [37].

Population Diversity and Generalizability Concerns

A crucial contradiction in current personalized HF research is the lack of population diversity in most studies, resulting in poor generalizability of findings. Genetic studies and digital health trials frequently feature homogeneous populations (often predominantly of European descent), severely limiting applicability across diverse ethnic, racial, and socioeconomic groups. Consequently, genetic risk scores, biomarker thresholds, and AI-driven algorithms developed in one population frequently fail validation in broader or more diverse patient cohorts, highlighting significant representational biases that undermine the core principle of personalization itself [38].

Healthcare Provider and Patient Acceptance

Another major obstacle to PM adoption is limited acceptance among clinicians and patients. Many healthcare providers remain hesitant to integrate genomic and digital tools into routine workflows, often due to limited training, perceived lack of clinical utility, time pressures, and insufficient decision-support infrastructure. On the patient side, receptiveness is similarly inconsistent, with concerns commonly revolving around data privacy, discomfort with constant monitoring, and uncertainty surrounding genetic information. Overcoming these attitudinal and behavioral challenges necessitates comprehensive educational interventions, effective communication strategies, and the development of patient- and clinician-centered tools grounded in real-world usability and trust-building principles [39].

Overall future directions in PM for HF management

Understanding the transformative potential of PM in HF management is both a compelling opportunity and a complex endeavor, demanding focused strategies, evidence-based exploration, and cohesive efforts across disciplines. Informed by a thorough evaluation of current literature, prevailing gaps, and critical limitations, a set of strategic priorities has proposed forthcoming research and support the practical translation of PM into clinical settings. The proposed future directions are summarized in Table 4.

**Table 4:** Future Directions with Actionable Steps of Personalized Medicine in Heart Failure The following authors have created this table: Elizabeth Caroline Palaparthi, Palle Aditya Reddy, Tanvi Padala, and Suresh Babu Sayana. RCTs: Randomized controlled trials

Key Future Direction	Possible Implementation Strategies
Robust clinical validation through large-scale trials	To conduct multi-center, international RCTs. Compare personalized management strategies against standard care. Clearly define endpoints (mortality, rehospitalization, quality of life) [40].
Standardization and consensus building	To establish expert panels for guideline development (e.g., ESC and AHA). Define universal thresholds for biomarkers and genetic variants. Develop standardized data integration protocols [13,41,42].
Multi-omic and integrated predictive modeling	To develop integrative platforms combining genomic, transcriptomic, proteomic, and metabolomic data. Utilize AI/machine learning algorithms for personalized risk assessment. Validate predictive models in diverse patient populations [25,26,43]
Addressing population diversity and equity	Design studies should include diverse ethnic and socioeconomic groups. Ensure genetic and technological tools are validated across diverse cohorts. Develop targeted strategies for equitable technology access and affordability [44].
Economic evaluations and cost-effectiveness studies	Conduct comprehensive health-economic analyses (QALYs, hospital admission rates, healthcare cost savings). Provide policymakers with robust economic evidence to guide resource allocation [25,26,43].
Ethical, legal, and social frameworks (ELSI)	Develop clear regulatory frameworks addressing genetic data privacy and patient autonomy. Include ethical assessments in clinical trials and technology deployment. Engage multidisciplinary stakeholders for policy formation [36].
Digital health integration and interoperability	Standardize interoperability protocols for integrating digital tools into electronic health records. Conduct pragmatic trials and pilot projects within clinical workflows. Address logistical and technical integration barriers systematically [27].
Education, training, and patient engagement initiatives	To provide comprehensive training for clinicians on PM approaches. Develop patient-centered educational resources to enhance understanding, adherence, and active participation. Incorporate user-centered design approaches for digital tools [44].

Robust Clinical Validation through Large-Scale Trials

Future research must prioritize the extent of prospective clinical trials, particularly large-scale RCTs, to provide definitive evidence on PM interventions like efficacy, safety, and cost-effectiveness. Such trials should explicitly compare personalized management strategies (e.g., genotype-guided therapy, integrated biomarker algorithms, digital interventions) against conventional approaches, measuring critical endpoints such as mortality, rehospitalization, quality of life, and patient adherence. Examples include trials validating pharmacogenomic prescribing algorithms (e.g., genotype-guided beta-blocker dosing) or comprehensive digital care platforms (remote monitoring and AI-driven predictive models). Nonetheless, global multi-center collaborations will also facilitate generalizability across diverse clinical and demographic settings [40].

Standardization and Consensus Building

To overcome fragmentation, efforts should aim toward international consensus and standardization in biomarker thresholds, genetic variant interpretation, and technology data integration. Professional societies, clinical guideline committees (ESC and AHA), and international consortia must collaboratively develop clear, uniform guidelines for integrating biomarkers, genetic data, and digital health tools into clinical practice. Establishing universally accepted standards would accelerate clinical translation and increase clinician confidence in adopting PM [13,41,42].

Multi-Omic and Integrated Predictive Modeling

One pivotal avenue for future exploration lies in integrating genomic, transcriptomic, proteomic, and metabolomic data into unified multi-omic platforms to enhance risk stratification and guide individualized treatment in HF. When paired with advanced artificial intelligence and machine learning methodologies, these comprehensive models offer the potential to forecast disease trajectories and patient-specific therapeutic responses accurately. Emerging research should focus on developing real-time, adaptive multi-omic monitoring systems that incorporate clinical parameters, including continuous data from wearable technologies, to optimize care on an ongoing basis. Demonstrating the clinical value and scalability of such integrative algorithms will require prospective validation across heterogeneous patient populations [25,26,43].

Addressing Population Diversity and Equity

A key priority moving forward is the pursuit of inclusive, demographically representative research that reflects the full spectrum of ethnic, racial, and socioeconomic diversity within HF populations. Purpose-built studies are needed to validate PM interventions across heterogeneous groups, enabling the identification of population-specific differences in genetic profiles, biomarker responses, and technology effectiveness that may impact clinical outcomes. Equally important is the advancement of equitable implementation strategies that deliberately address systemic barriers to access and cost to ensure that personalized approaches contribute to narrowing, rather than widening, existing disparities in healthcare delivery and outcomes [44].

Economic Evaluations and Cost-effectiveness Studies

Future research should emphasize comprehensive health economic analyses and cost-effectiveness studies to demonstrate PM interventions' economic sustainability and societal value. Detailed evaluations, including quality-adjusted life-years (QALYs), hospital admission reductions, and long-term healthcare costs, will provide essential evidence for policymakers and healthcare administrators. This will support strategic investment decisions and foster the integration of PM into healthcare systems globally, particularly in resource-constrained settings.

Ethical, Legal, and Social Frameworks (ELSI)

As genetic testing and continuous monitoring technologies expand, developing robust ELSI to protect patient privacy, data security, and autonomy is imperative. Clear regulatory guidelines on genetic data ownership, sharing, informed consent, and patient rights must accompany technological advancements. Future research should include bioethical studies assessing patient and clinician perspectives, societal attitudes, and implications of PM technologies, facilitating ethically sound, patient-centered clinical adoption [36].

Digital Health Integration and Interoperability

Seamless integration of digital health technologies (wearable devices, remote monitoring platforms, AI tools) into routine clinical practice represents another critical future goal. Efforts should prioritize developing standardized interoperability protocols and integrating these data into existing EHR systems. Real-world pilot programs and pragmatic trials testing digital health platforms' integration with existing clinical workflows can identify logistical barriers and solutions, paving the way for widespread adoption [27].

Education, Training, and Patient Engagement Initiatives

To foster successful implementation, future initiatives must include extensive education, training, and awareness programs tailored to clinicians, healthcare providers, and patients. Education programs should address PM concepts, genetic counselling, interpretation of biomarker results, use of digital monitoring tools, and ethical considerations. Patient engagement initiatives should empower individuals with knowledge and tools to actively participate in their care, emphasizing transparency, user-friendliness, and patient-centered design to enhance acceptance and adherence [44].

**Figure 1:**
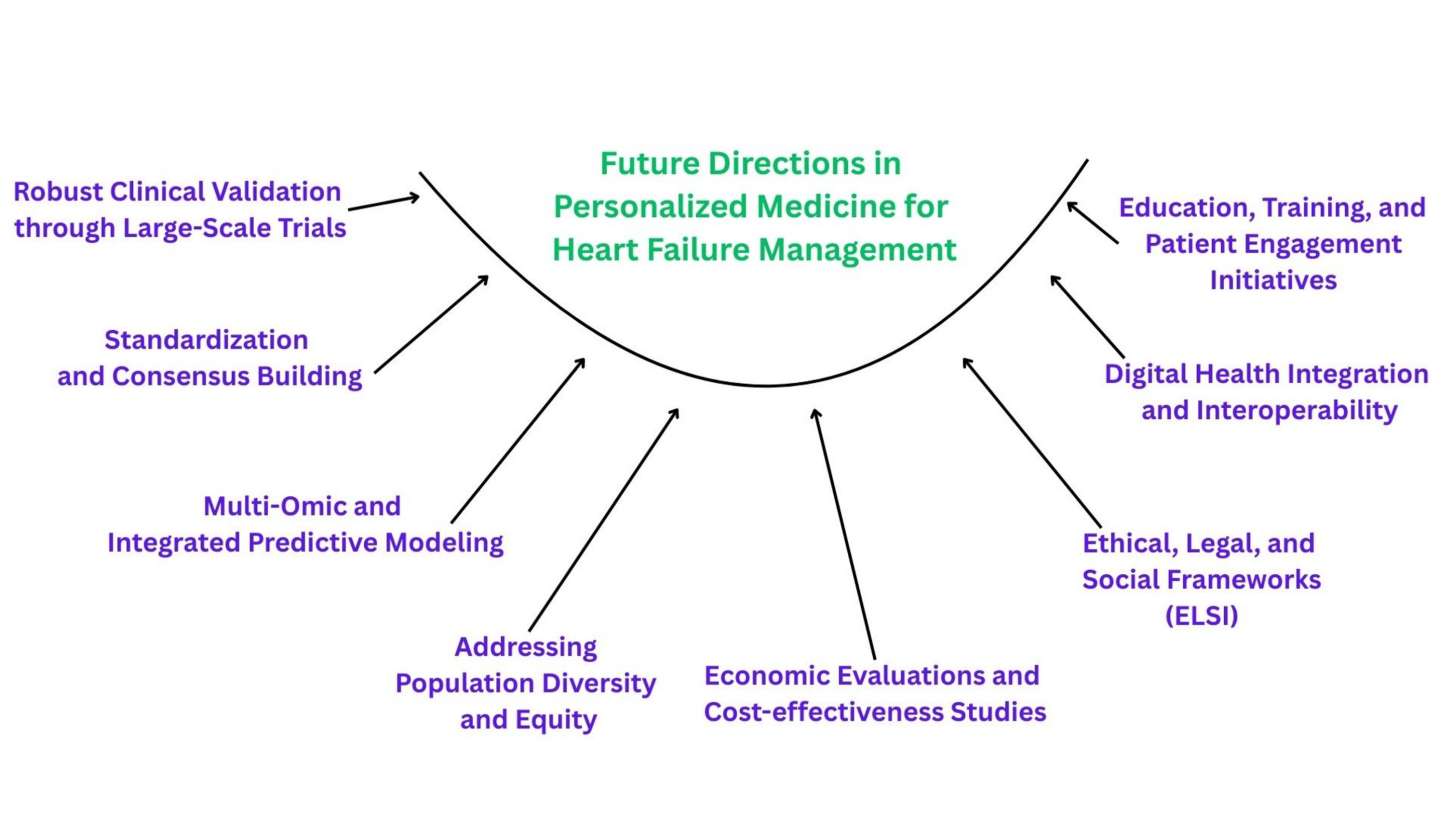
Future Directions with Actionable Steps for Personalized Medicine in Heart Failure This image was created by the authors using PowerPoint tools (Microsoft® Corp., Redmond, WA, USA).

**Figure 2:**
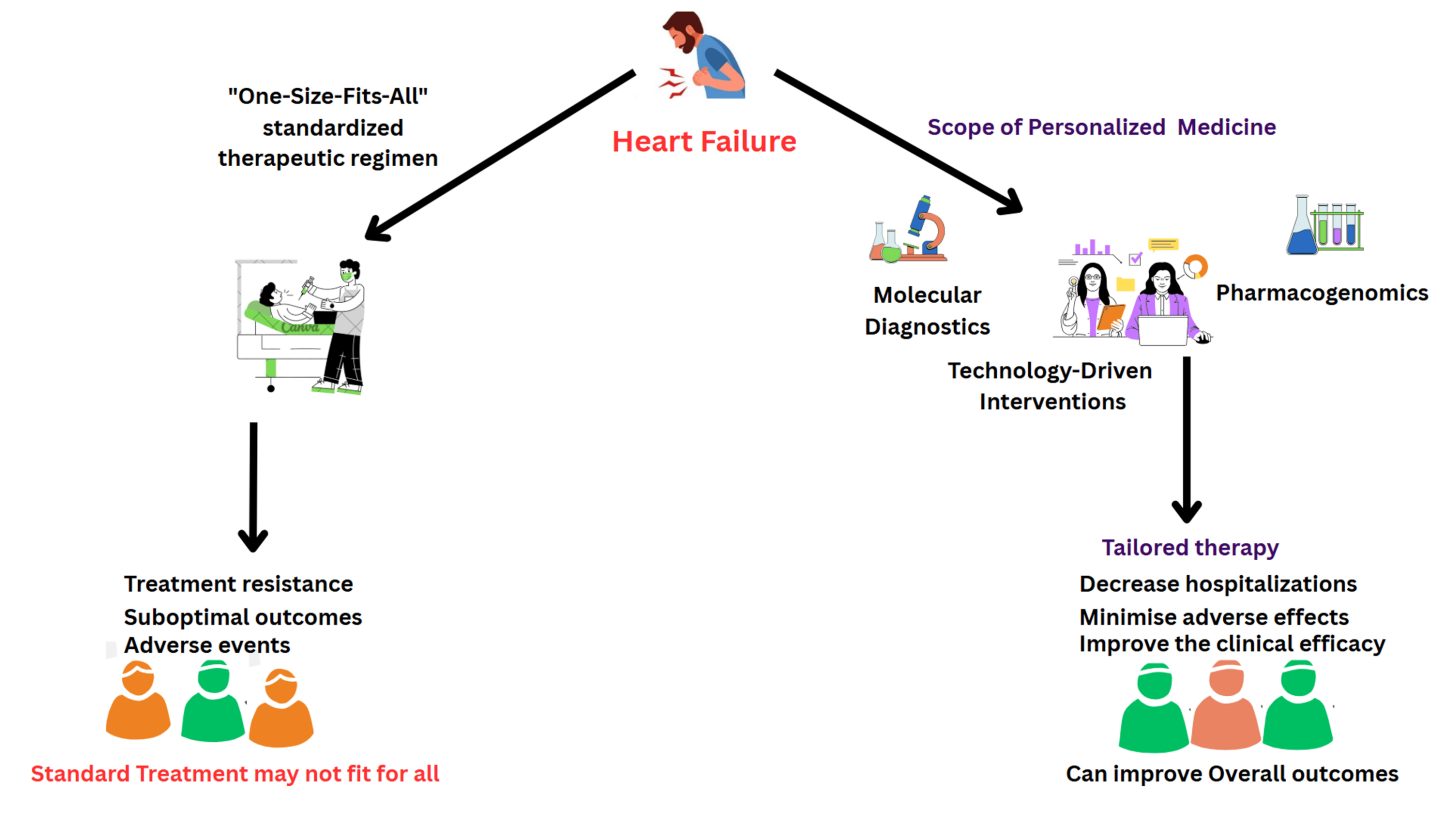
The Scope of Personalized Medicine in Heart Failure Management The author Tambi Medabala created this image using Canva.com. The figure depicts the conventional “one-size-fits-all” approach to HF treatment with a PM strategy. Standardized regimens often lead to suboptimal outcomes, treatment resistance, and adverse effects due to patient heterogeneity. In contrast, the PM pathway leverages molecular diagnostics (e.g., biomarker panels, multi-omic profiling), pharmacogenomics (e.g., genotype-guided therapy), and technology-driven interventions (e.g., implantable sensors, AI-based remote monitoring) to enable tailored therapies. This integrated approach aims to reduce hospitalizations, minimize adverse effects, and improve clinical efficacy and overall patient outcomes. HF: Heart failure; PM: personalized medicine

Figure 1 presents key strategic priorities to advance the clinical application of PM in HF. These include large-scale clinical validation, standardization efforts, multi-omic and predictive modelling, and digital health integration. Emphasis is also placed on addressing population diversity, cost-effectiveness, ELSI, and patient-centered engagement. Together, these domains form a comprehensive roadmap toward evidence-based, equitable, and scalable personalized HF management. Figure 2 illustrates the scope of personalized medicine in HF management, highlighting key elements such as molecular diagnostics, pharmacogenomics, and technology-driven interventions.

## Conclusions

This review emphasizes the transformative role of PM in HF management by integrating molecular diagnostics, pharmacogenomics, and technology-driven interventions to improve patient-specific outcomes significantly. It clearly illustrates a departure from traditional generalized approaches, advocating precise treatment targeting, reduced adverse events, minimization of hospitalization, and improved quality of life. Nonetheless, significant implementation challenges persist, including methodological limitations, standardization gaps, ethical concerns, economic constraints, and disparities in healthcare accessibility and population representation, which currently restrict widespread clinical adoption. Addressing these barriers requires laborious validation through large-scale RCTs, standardized biomarker and genetic methodologies, interoperable digital health systems, and extensive provider and patient education. These strategies form a coherent roadmap toward achieving effective, equitable, and sustainable personalized HF management.
